# Comparison between cigarette smoke-induced emphysema and cigarette smoke extract-induced emphysema

**DOI:** 10.1186/s12971-015-0033-z

**Published:** 2015-03-25

**Authors:** Zhi-Hui He, Ping Chen, Yan Chen, Sheng-Dong He, Ji-Ru Ye, Hong-Liang Zhang, Jun Cao

**Affiliations:** Intensive Care Unit, the Second Xiangya Hospital, Central-South University, Changsha, Hunan 410011 China; Department of Respiratory Medicine, The Second Xiangya Hospital, Central-South University, Changsha, Hunan 410011 China; Division of Respiratory Disease, Department of Internal Medicine, The Second Xiangya Hospital, Central-South University, Changsha, Hunan 410011 China; Department of Respiratory Medicine, Hunan Provincial People’s Hospital, Changsha, Hunan 410005 China

**Keywords:** Animal, Chronic obstructive pulmonary disease (COPD), Emphysema, Model

## Abstract

**Background and objective:**

Emphysema is the main pathological feature of COPD and also is the focus of the related research. Although several emphysema animal models have been established, exact comparison of findings is seldom. The present study aimed to compare cigarette smoke (CS) exposure-induced emphysema model and intraperitoneal injection of cigarette smoke extract (CSE)-induced emphysema model to evaluate the effectiveness of the two different modeling methods.

**Methods:**

Six-week-old male C57BL/6 J mice were used and randomly divided into two groups: CS exposure and intraperitoneal injection of CSE. Each group was subdivided into two subgroups: control and CS or CSE. Lung function, mean linear intercept (MLI), destructive index (DI), apoptotic index (AI), total and differential cells count in broncholavolar lavage fluid (BALF), SOD and IL-6 concentration in serum were measured.

**Results:**

Compared with their respective controls, lung function was significantly decreased in CS and CSE groups (*P* < 0.01); MLI, DI, and AI of lung tissue were significantly higher in CS and CSE groups (*P* < 0.01); total number of leukocytes, the number and percentage of neutrophils (NEUs), and the number of macrophages (MAC) in BALF were significantly higher in CS and CSE groups (*P* < 0.01); SOD concentration in serum was significantly decreased in CS and CSE groups (*P* < 0.01); IL-6 concentration in serum was significantly increased in in CS and CSE groups (*P* < 0.01). There was no significant difference between CS group and CSE group in any of the parameters described above.

**Conclusions:**

Both CS exposure and intraperitoneal injection of CSE could induce emphysema and the effectiveness of the two different modeling methods were equal.

## Introduction

Chronic obstructive pulmonary disease (COPD) is a progressive chronic respiratory disease of human beings characterized by not fully reversible airflow limitation. It is mainly caused by cigarette smoke and is the fourth leading cause of death worldwide. According to the World Health Organization, its prevalence will double by 2020 [1] and it will become the third leading cause of death worldwide [2]. But the mechanism of COPD is not completely illuminated. Direct research on human body was limited because of the anthropic ethics. Therefore, research on animal model is particularly important. Emphysema is the main pathological feature of COPD and also is the focus or hotspot of research which focuses on the mechanism of COPD.

Although several emphysema animal models have been established, exact comparisons of findings from various groups are difficult because different methods, different types of cigarettes, different doses of cigarette smoke, instruments, exposure protocols and a wide variety of animals are used. Some of the models were insufficient in quantitative evaluation. Smoking is the most important risk factor for emphysema. Cigarette smoke (CS) is a mixture of more than 4,000 different chemical compounds, such as free radicals, toxins, and electrophiles, etc. [3,4]. The CS extract (CSE) contains nearly all of the compounds inhaled by smokers. In resent years, animal model of emphysema was also established by intraperitoneal injection of CSE [5,6]. The present study aim to investigate whether the effectiveness of CS exposure and intraperitoneal injection of CSE on emphysema were equal. We used the same cigarettes and animals, and compared CS exposure-induced emphysema and intraperitoneal injection of CSE-induced emphysema in lung function and histomorphology, apoptosis of alveolar septum cells, total and differential cell counts in broncholavolar lavage fluid (BALF), SOD and IL-6 concentrations in serum.

## Materials and methods

### Animals

Forty-eight 6-week-old male C57BL/6 J mice (Wt: 20.26 ± 2.34 g) were randomly enrolled in this study and randomly divided into two groups: CS exposure group (group 1) and intraperitoneal injection of CSE group (group 2). Each group was further divided into two subgroups: control (group N) and CS or CSE (group C), named N1, C1, N2, C2 respectively (n = 10 per subgroup). All animals were provided by Shanghai Laboratory Animal Center of Chinese Academy of Sciences (SLACCAS, Shanghai, China) and fed in a clean unit at 23°C ~ 25°C, 50% ~ 60% humidity, 12 hours (h) rhythm of light and dark. They were provided free access to water and food. The Second Xiangya Hospital Experimental Animal Center of Central South University was responsible for feeding.

The study was approved by the Institutional Review Board of Central-South University and conformed to the guiding principles for research involving animals and human beings [7].

### Preparation of CSE

CSE was prepared using a technique described previously [8] with some modification. Briefly, one non-filtered Fu-Rong cigarette (Tar: 13 mg, Nicotine: 1.0 mg, Carbon Monoxide: 14 mg/cigarette, China Tobacco Hunan Industrial Co., Ltd., Changsha, China) was burned and the smoke passed through 4 ml of phosphate-buffered saline (PBS) by connecting to a vacuum pump with a constant pressure of −0.1Kpa. This solution was used for intraperitoneal injection after filtering through a filter with 0.22-μM pores (Fisher Scientific International Inc., Hampton, NH, USA) to remove particles and bacteria. The solution was prepared fresh for each injection.

### Emphysema models

#### (1) Cigarette smoke exposure

The glass box used for modeling was made by ourselves and with the size of (69 cm × 47 cm × 38 cm), round hole with 1 cm diameter at a density of 1 hole per 100 cm^2^ on the lid and 1 hole per 250 cm^2^ on four sides of the box. In the box, a partition with the same size holes at a density of 1 hole per 6 cm^2^ was placed in the middle of the box to divided it into two parts: the lower for cigarettes burning, and the upper for animal exposure to the smoke. Firstly, five cigarettes were burned at the same time with the smoke lasting for 15 min. Secondly, the box was opened to let the animals rest for 5 min. Then the first step was repeated. This process was referred to as one cycle of CS exposure. Mice were exposed for 2 cycles/day, 6 days/week for 12 weeks. The control group was fed in Hospital Experimental Animal Center of Central South University.

#### (2) Intraperitoneal injection of CSE

The emphysema model was established as previously described [9]. The total experimental period was four weeks. On day 1, 12 and 23, animal in control group was given an intraperitoneal injection of 0.3 ml/20 g PBS, and animal in CSE group was given an intraperitoneal injection of 0.3 ml/20 g CSE-PBS. On day 29, the mice were disposed for lung function measurement, blood collection, broncholavolar lavage (BAL) and histomorphological detection of lung tissue.

### Lung function measurement

Lung function was measured using small animal spirometer (PLY3211 system, Buxco Electronics, USA) as previously described with a minor modification [10]. Briefly, the mouse was weighed, anesthetized by intraperitoneal injection of 10% chloral hydrate (3 ml/kg BW) and tracheostomized. The trachea was cannulated, and the cannula was connected to a computer-controlled small animal spirometer. Airway resistance (Raw), lung dynamic compliance (Cdyn), peak expiratory flow (PEF) and inspiratory time/expiratory time (Ti/Te) were measured.

### Histomorphology detection of lung tissue

After lung function measurement, animal was sacrificed by overdose of anesthetics. The lower left lobes of lungs were inflated with 4% paraformaldehyde at a constant pressure of 25 cm H_2_O, then fixed with 4% paraformaldehyde for 24 h [10]. Fixed lung was embedded in paraffin (Sigma, USA) and sectioned into 4-μm sections. The slices were stained with hematoxylin and eosin (H & E) (Sigma, USA). Emphysema was quantified based on the measurement of the mean linear intercept (MLI) and destructive index (DI). The MLI and DI were measured as previously described [11]. Briefly, the MLI was measured by dividing the length of a line drawn across the lung section by a total number of intercepts counted within this line. The DI was calculated by dividing the defined destructive alveoli by the total number of alveoli.

### Apoptosis assay of alveolar septum cells

Terminal deoxynucleotidyl transferase-mediated dUTP nick end labeling (TUNEL) was performed to label the DNA-damaged cells using In Situ Cell Death Detection Kit (Roche Diagnostics, Mannheim, Germany) following the manufacturer’s instructions. The apoptotic index (AI) was calculated as the percentage of TUNEL-positive nuclei.

### Total and differential cells count in broncholavolar lavage fluid (BALF)

The left lung was clamped and the right lung was flushed 3 times with 1.0 mL PBS. BALF was pooled and the total volume was recorded. About 90% of the instilled PBS was collected from each animal. The BALF was immediately centrifuged for 10 minutes at 1500 rpm (400 × *g*) and 4°C, and the cells were separated for counting. Cells were stained with Wright-Giemsa stain according to the manufacturer’s instructions (Beyotime Institute of Biotechnology, China). Counts and differentials were manually determined using a standard hemocytometer. (Beyotime Institute of Biotechnology, China). Wright-Giemsa-stained slides were examined in a random sequence. 400 leukocytes were counted on each slide and measured by high microscopy at a magnification of × 1000. The percentages of neutrophils (NEU) and macrophages (MAC) were analyzed.

### Measurement of SOD and IL-6 concentrations in serum

The concentrations of SOD and IL-6 in serum were measured with ELISA kits (R & D Systems, Pittsburg, PA, USA and Jingmei Biotech Co. Ltd., Shenzhen, China, respectively) according to the manufacturers’ instructions.

### Statistical analysis

Analyses were performed using SPSS for Windows 16.0 (SPSS Inc., Chicago, IL, USA). All data were expressed as means ± standard deviation. Analysis of differences among groups were performed using analysis of variance (one-way ANOVA), followed by post-hoc analysis as appropriate. Values of *P* < 0.05 were considered statistically significant.

## Results

### Lung function

Raw was increased in mice induced by CS or CSE when compared with the respective controls (*P* < 0.01). Cdyn, PEF and Ti/Te were decreased in mice induced by CS or CSE when compared with the respective controls (*P* < 0.01). There was no significant difference between CS group and CSE group in Raw, Cdyn, PEF or Ti/Te (*P* > 0.05) (Table 1).

**Table 1:** **Lung function**

	**CS (group 1)**		**CSE (group 2)**	
	**N1 (n = 10)**	**C1 (n = 10)**	**N2 (n = 10)**	**C2 (n = 10)**
Raw	0.42 ± 0.06	1.92 ± 0.36★	0.45 ± 0.07	2.05 ± 0.39★
(cmH_2_OmL^−1^min^−1^)				
Cdyn (mL/cmH_2_O)	2.72 ± 0.51	1.05 ± 0.22★	2.25 ± 0.37	1.02 ± 0.23★
PEF (mL/s)				
Ti/Te	6.55 ± 1.05	4.37 ± 0.76★	6.06 ± 0.71	3.91 ± 0.77★
	0.85 ± 0.14	0.60 ± 0.11★	0.88 ± 0.11	0.56 ± 0.11★

Lung function was measured 12 weeks after the start of CS exposure (group 1) or 29 days after the start of intraperitoneal injection of CSE (group 2). CS, cigarette smoke; CSE, cigarette smoke extract; N1, controls of CS exposure group; C1, CS exposure group; N2, controls of intraperitoneal injection of CSE group; C2, intraperitoneal injection of CSE group; Raw, airway resistance; Cdyn, lung dynamic compliance; PEF, peak expiratory flow; Ti, inspiratory time; Te, expiratory time. Valus are means ± standard deviation, ★*P* < 0.01 compared with the respective group N.

### Histomorphological changes of lung tissues

**Figure 1:**
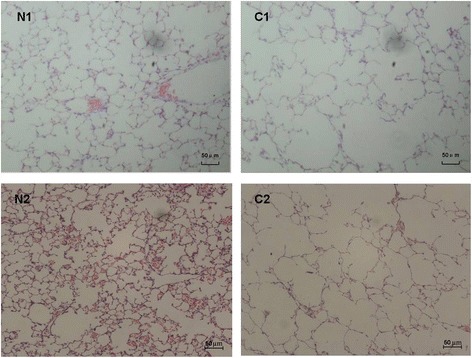
**Lung histomorphology of C57BL/6 J mice induced by CS exposure and intraperitoneal injection of CSE (×100).** Lung tissues of C57BL/6 J mice induced by CS exposure **(C1)** exhibited enlarged alveolar space, thinner alveolar septum, and destroyed alveolar wall when compared with those of the controls **(N1)**. Lung tissues of C57BL/6 J mice induced by intraperitoneal injection of CSE **(C2)** also exhibited enlarged alveolar space, thinner alveolar septum, and destroyed alveolar wall when compared with those of the controls **(N2)**. CS, cigarette smoke; CSE, cigarette smoke extract.

**Figure 2:**
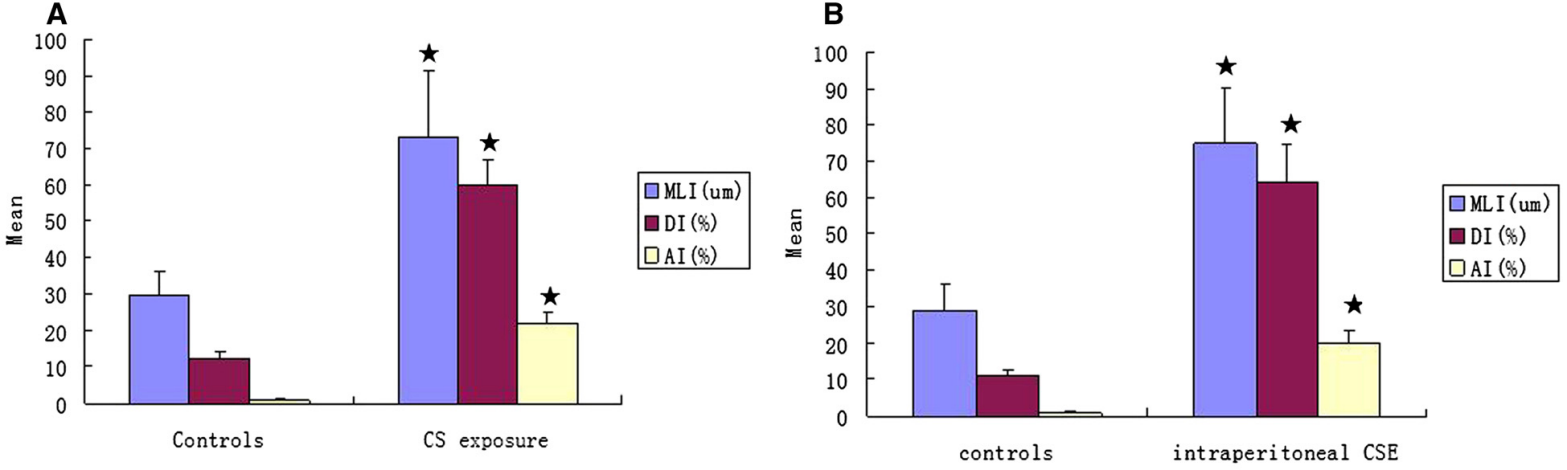
**MLI, DI, and AI in C57BL/6 J mice induced by CS exposure (A) and that induced by intraperitoneal injection of CSE (B).** MLI, mean linear intercept; DI, destructive index; AI, apoptotic index; CS, cigarette smoke; CSE, cigarette smoke extract. ★*P* < 0.01 compared with controls.

Lung tissue of mice induced by CS or CSE exhibited enlarged alveolar space, thinner alveolar septum and destroyed alveolar wall (Figure 1). The MLI and DI in mice induced by CS or CSE were increased when compared with the respective controls (*P* < 0.01) (Figure 2). There was no significant difference between CS group and CSE group in MLI or DI (*P* > 0.05).

### Apoptosis of alveolar septum cells

**Figure 3:**
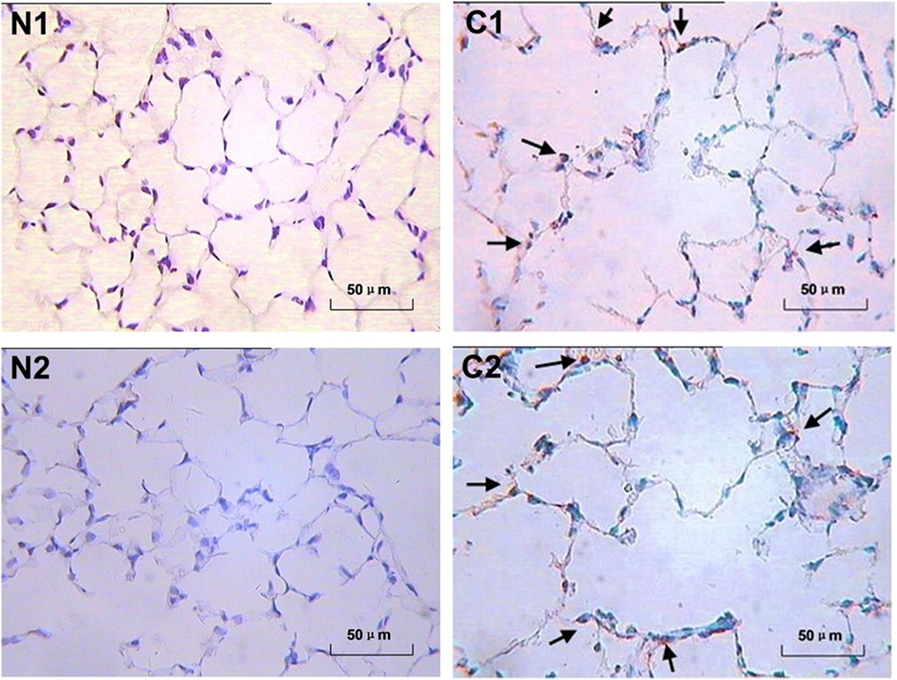
**Apoptosis of alveolar septum cells in C57BL/6 J mice induced by CS exposure and that induced by intraperitoneal injection of CSE (×400).** The number of apoptotic alveolar septum cells in C57BL/6 J mice induced by cigarette smoke exposure **(C1)** was increased when compared with the controls **(N1)**. The number of apoptotic alveolar septum cells in C57BL/6 J mice induced by intraperitoneal injection of cigarette smoke extract **(C2)** was also increased when compared with the controls **(N2)**.

The number of apoptotic alvelolar septum cells in mice induced by CS or CSE was increased when compared with the respective controls (Figure 3). The AI of alvelolar septum cells in mice induced by CS or CSE was higher than that of the respective controls (*P* < 0.01) (Figure 3). There was no significant difference between CS group and CSE group in AI (*P* > 0.05).

### Total and differential cells count in BALF

As shown in Table 2, the number of total cells, MAC, NEU and the percentage of NEU (NEU%) in mice induced by CS or CSE were higher than those in their respective controls (*P* < 0.01). There was no statistic difference in any parameters described above between CS group and CSE group (*P* > 0.05) (Table 2).

**Table 2:** **Total and differential cells count in BALF**

	**CS (group 1)**		**CSE (group 2)**	
	**N1 (n = 10)**	**C1 (n = 10)**	**N2 (n = 10)**	**C2 (n = 10)**
Total cells (×10^8^/L)	1.46 ± 0.25	5.35 ± 1.05★	1.46 ± 0.23	5.07 ± 1.01★
MAC (×10^8^/L)	1.07 ± 0.20	3.34 ± 0.66★	1.31 ± 0.20	3.51 ± 0.56★
MAC%(%)				
NEU (×10^7^/L)	71.82 ± 13.85	59.15 ± 10.30★	68.03 ± 14.76	51.07 ± 10.62★
NEU%(%)	1.35 ± 0.20	13.01 ± 2.23★	1.45 ± 0.19	13.76 ± 2.38★
	9.85 ± 1.75	19.95 ± 3.46★	9.67 ± 1.59	21.49 ± 3.95★

BALF, broncholavolar lavage fluid; CS, cigarette smoke; CSE, cigarette smoke extract; N1, controls of CS exposure group; C1, CS exposure group; N2, controls of intraperitoneal injection of CSE group; C2, intraperitoneal injection of CSE group; MAC, macrophage; NEU, neutrophil. Valus are means ± standard deviation, ★*P* < 0.01 compared with the respective group N.

### SOD and IL-6 concentrations in serum

**Figure 4:**
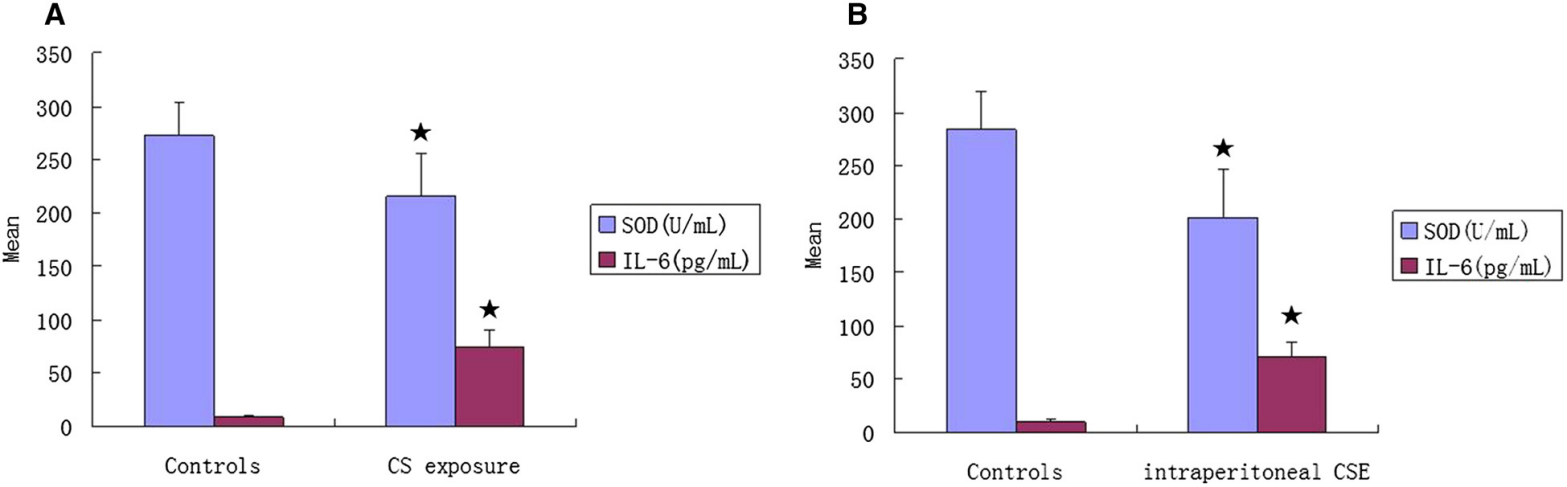
**SOD and IL-6 concentrations in serum of C57BL/6 J mice induced by CS exposure (A) and that induced by intraperitoneal injection of CSE (B).** SOD, superoxide dismutase; IL-6, interleukin-6; CS, cigarette smoke; CSE, cigarette smoke extract. ★*P* < 0.01 compared with controls.

The SOD concentration in the serum of mice induced by CS (215.91 ± 39.44 U/mL, *P* < 0.01) or CSE (200.93 ± 45.86 U/mL, *P* < 0.01) were lower than those in their respective controls (272.33 ± 31.26 U/mL and 284.08 ± 36.09 U/mL, respectively). There was no significant difference in SOD concentration (*P* > 0.05) between CS group and CSE group. The IL-6 concentration in the serum of mice induced by CS (73.54 ± 16.02 pg/mL, *P* < 0.01) or CSE (70.74 ± 13.43 pg/mL, *P* < 0.01) were higher than those in their respective controls (8.88 ± 1.56 pg/mL and 10.03 ± 1.87 pg/mL, respectively). There was no significant difference in IL-6 concentration (*P* > 0.05) between CS group and CSE group (Figure 4).

## Discussion

The present study showed that both CS exposure and intraperitoneal injection of CSE could induce emphysema manifested in decreased lung function, enlarged alveolar space, destroyed alveolar wall, increased apoptosis in alveolar septum cells and increased inflammatory cells in BALF in mice. Meanwhile, the decreased SOD concentration and increased IL-6 concentration in the serum could be observed in both CS exposure-induced emphysema and intraperitoneal injection of CSE-induced emphysema. More importantly, the changes described above induced by the two different methods were the same.

Pulmonary function is an important criterion for the evaluation of emphysema model. There was global strategy for the diagnosis, classification of COPD in human [12], but no for animal emphysema or COPD. The present study showed that mice induced by CS or CSE exhibited decreased lung function. Pulmonary function tests were considered to be less sensitive than morphometry and might detect only more severe degrees of airways remodeling or parenchymal destruction. Mild emphysema might have normal lung function [13]. These viewpoints indicated that the emphysema models in the present study might represent severe cases of emphysema.

Ochs M. suggested that quantitative assessment of micro-structure was the only way to reliably demonstrate the presence of emphysematous alterations [14]. According to the American Thoracic Society, emphysema was defined as “abnormal, permanent enlargement of the airspaces distal to the terminal bronchiole, accompanied by destruction of their walls” [15]. This widely accepted definition was fulfilled in both CS exposure-induced emphysema and intraperitoneal injection of CSE-induced emphysema.

Apoptosis of alveolar septal cells plays an important role in the development of emphysema [16]. Apoptotic index (AI) reflects apoptosis status. In the present study, AI of alveolar septum cells in emphysema animals whether induced by CS exposure or intraperitoneal injection of CSE were significantly increased.

Our previous study showed that the inflamed airways of COPD patients contained several inflammatory cells including NEU and MAC [17]. In the present study, the increased inflammatory cells were also observed in BALF of all emphysema animal, whether induced by CS exposure or intraperitoneal injection of CSE.

Oxidative stress is an important mechanism in the pathogenesis of COPD. When the resident antioxidants are insufficient or fail to upregulate sufficiently to neutralize an increased oxidant burden, oxidative stress occurs. ROS contributes to a variety of adverse consequences, including cell apoptosis, inflammatory responses, and impaired tissue repair, and all of these processes are intimately associated with oxidative stress [18]. SOD could decrease markers of oxidative stress in patients with emphysema [19]. Extracellular SOD could protect against pulmonary emphysema and lung inflammation induced by cigarette smoke by decreasing oxidative fragmentation of the extracellular matrix [20]. IL-6 is a pro-inflammatory mediator [21] and is regarded as a COPD candidate gene [22]. IL-6 could promote the development of pulmonary emphysema associated with apoptosis in mice [23]. The important role of IL-6 in the pathogenesis of emphysema was further suggested by clinical studies demonstrating the elevated systemic IL-6 concentrations in patients with emphysema [17,24]. In the present study, the decreased SOD concentration and increased IL-6 concentration in the serum were confirmed in the emphysema models whether induced by CS exposure or intraperitoneal injection of CSE.

To date, a large variety of emphysema animal models have been developed in various species including dogs, monkey, pigs, sheep, rabbits, guinea pigs, rats and mice [25-31]. Emphysema animal model has been established by exposure to smoking [32], intranasal instillation of elastase [33], intranasal instillation of LPS [34], exposure to sulfur dioxide [35], inhalation of ovalbumin dry powder [36], intravenous injection of hyaluronidase [37], genetic manipulation [38], and intraperitoneal injection with xenogeneic endothelial cells [39], and intraperitoneal injection with CSE [6]. Among the emphysema animal models, mice have become very popular for experiment because their genome are much more like human being’s than many other animals [40] and they offer the advantages of low cost, extensive gene/protein sequence/antibody availability, and, most important, the availability of numerous naturally occurring mouse strains with different reactions to smoke. But the study on the comparison of models is seldom.

Cigarette smoking is by far the most important risk factor for emphysema and COPD. CS induces significant increases in the generation of reactive oxygen species (ROS) [41]. ROS contributes to a variety of adverse consequences including cell apoptosis, inflammatory responses and impaired tissue repair [18]. Augmented apoptosis, impaired efferocytosis and abnormal tissue remodeling contribute to the chronic inflammatory response and tissue destruction in emphysema and COPD [42]. Interestingly, the present study showed that there was no significant difference between mice induced by CS exposure and that induced by intraperitoneal injection of CSE in lung function, histomorphology, apoptosis of alveolar septum cells, total and differential inflammatory cells count in BALF, SOD and IL-6 concentrations in serum. These results suggested that the modeled effects of the two different modeling methods, CS exposure and intraperitoneal injection of CSE, were equal.

CS exposure was looked as the traditional method of long-term modeling of emphysema. The various times spent on modeling might be due to the different kind of cigarette, different exposure mode, duration and frequency, different smoke density, different species and age of animals and so on. Because of the long modeling time, inconsistence and unstability, researchers have constantly explored new modeling methods. As the surrogate of CS, CSE could be considered to play the same role as CS in emphysema and COPD. CSE could decrease the function of endothelial progenitor cells (EPCs) [43], induce the apoptosis of pulmonary endothelial cells [44]. Intraperitoneal injection of CSE is a relatively new method of short-term modeling of emphysema, which was firstly reported by Taraseviciene-Stewart et al. in 2007 [45]. Taraseviciene-Stewart et al. reported the emphysema model induced by intraperitoneal injection with xenogeneic endothelial cells in 2005 [39], and then used CSE instead of xenogeneic endothelial cells. The mechanism of intraperitoneal CSE-induced emphysema model is under disscusion. Taraseviciene-Stewart et al. hypothesized that CSE could act as an antigen triggering an immune response as well as oxidative stress that induced emphysema [45]. Zhang Y et al. thought that the mechanism of intraperitoneal CSE-induced model was link to apoptosis of pulmonary vascular endothelium. Intraperitoneal injection of CSE, corresponding to systemic delivery of CSE, reduced the biological antioxidant activity in BALF causing direct alveolar septum cells apoptosis and endothelium damage, which allowed inflammatory cells to infiltrate in the lung tissue [9]. It is unclear whether all pathophysiologically relevant mechanisms in this model are shared with the conventional model in which cigarette smoke is inhaled over a period of several months. The problem whether CSE impairs lung tissue targetedly making inflammatory cells homing in focus or the systemic inflammatory cells induced by CSE infiltrate in the lung tissue through the impaired endothelium needs further study. Systemic inflammatory process could affect lung tissue directly by releasing cytokines and chemokines or indirectly by activating lung inflammatory cells. More than one pathway might be operational at one time.

Although mice and humans share many basic physiological processes, specific differences in lung structure, function and immunology between humans and mice have to be taken into consideration. Even within mice, different strains exhibit different sensitivities to the development of emphysema [46]. None of the models reproduces the exact changes seen in humans, each has its own advantages and disadvantages. Although emphysema induced by intraperitoneal injection of CSE was similar to COPD in human in lung function, inflammatory cells in BALF, histomophology of lung tissue, we called it cautiously the “emphysema model”, not “COPD model”, on account of the modeling method. In fact, it is impossible for human to “smoke” by intraperitoneal injection of CSE. Therefore, it would not be known that whether this method could induce emphysema or even COPD in human.

## Conclusion

The present study demonstrated that emphysema with decreased lung function, enlarged alveolar space, destroyed alveolar wall, apoptosis of alveolar septum cells, chronic inflammation in lung, decreased SOD concentration and increased IL-6 concentration in serum could be duplicated in mice induced by CS exposure or intraperitoneal injection of CSE. More importantly, the effectiveness of the two different modeling methods were equal.

## Abbreviations

AI: Apoptotic index
BALF: Broncholavolar lavage fluid
Cdyn: Lung dynamic compliance
COPD: Chronic obstructive pulmonary disease
CS: Cigarette smoke
CSE: Cigarette smoke extract
DI: Mean linear intercept
EPCs: Endothelial progenitor cells
IL-6: Interleukin-6
MAC: Macrophage
MLI: Mean linear intercept
NEU: Neutrophil
PEF: Peak expiratory flow
Raw: Airway resistance
ROS: Reactive oxygen species
SOD: Superoxide dismutase
Te: Expiratory time
Ti: Inspiratory time
